# Clinical characteristics and fatal outcomes of hypertension in patients with severe COVID-19

**DOI:** 10.18632/aging.104019

**Published:** 2020-11-16

**Authors:** Xiaocheng Cheng, Guoqiang Cai, Xuesong Wen, Lei Gao, Dan Jiang, Min Sun, Shu Qin, Jianzhong Zhou, Dongying Zhang

**Affiliations:** 1 Department of Cardiology, The First Affiliated Hospital of Chongqing Medical University, Chongqing 400016, China; 2 Department of Emergency, Traditional Chinese Medicine Hospital Dianjiang Chongqing, Chongqing 408300, China

**Keywords:** COVID-19, hypertension, ACEI/ARB, mortality, cardiac injury

## Abstract

The aim of this study is to investigate clinical characteristics and fatal outcomes of hypertension as well as the role of angiotensin-converting enzyme inhibitors/angiotensin receptor blockers (ACEI/ARB) use in patients with severe coronavirus disease 2019 (COVID-19). A total of 220 (female: 51.8%) patients with severe COVID-19 were included. The mean age of included patients was 59.5 years and 70 (31.8%) patients had a history of hypertension. There were 23 patients (32.9%) receiving ACEI/ARB therapy. Patients with hypertension were older and had more comorbidities, and were more likely to suffer from severe inflammatory response and acute cardiac injury. Moreover, patients with hypertension were associated with significantly higher risk of in-hospital mortality than patients without hypertension. After adjustment of potential confounders, the independent correlation was still observed. In addition, ACEI/ARB users were associated with lower level of high-sensitivity cardiac troponin I and creatinine kinase–myocardial band, and lower risk of acute cardiac injury than ACEI/ARB non-users. In conclusion, patients with hypertension were more likely to suffer from severe inflammatory response, acute cardiac injury and had high risk of in-hospital mortality in severe COVID-19. The use of ACEI/ARB may protect patients with COVID-19 from acute cardiac injury.

## INTRODUCTION

The coronavirus disease 2019 (COVID-19) has become a worldwide pandemic [1–4]. Some studies have reported information on the epidemiology and clinical features of COVID-19, which suggested that COVID-19 can cause acute respiratory distress syndrome (ARDS), multiple organ dysfunction syndrome and 2 to 21% risk of death [1–4, 5]. It has been shown that hypertension was one of the most distinctive comorbidities in COVID-19 infection. The study by Guan and colleagues enrolled 1099 patients with confirmed COVID-19, 23% of whom had a history of hypertension in severe cases [1]. Richardson et al. reported that hypertension was the most common comorbidities in COVID-19, reaching to 56.6% [4]. However, it is unclear about the clinical characteristics of hypertension infected COVID-19, and whether hypertension is associated with poor clinical outcomes is also less clear.

Notably, hypertension may frequently be treated with angiotensin converting enzyme inhibitors (ACEI)/angiotensin receptor blockers (ARB) [6–7]. But the administration of ACEI/ARB in COVID-19 was controversial. Some researchers have put forward the hypothesis that ACEI/ARB might become a potential risk factor for fatal COVID-19 by up-regulating the expression of angiotensin-converting enzyme 2 (ACE2) [8–9]. In view of the overwhelming evidence of mortality reduction in cardiovascular disease, however, some investigators suggested that ACEI/ARB therapy should be maintained or initiated in patients with hypertension according to current guidelines [10].

Up to date, there are limited clinical studies evaluating the safety of ACEI/ARB in the treatment of COVID-19 with hypertension. The purpose of this study was to investigate clinical characteristics and outcomes of hypertension as well as the role of ACEI/ARB in severe COVID-19.

## RESULTS

### Baseline characteristics and laboratory findings

The study initially enrolled 237 (51.8% female) consecutive patients with severe COVID-19. We excluded 14 discharged patients and 3 patients who died because of incomplete data, leaving 220 patients for final analysis. The median age of included patients was 59.5 years (range 24 to 94 years). Sixty-two (31.8%) patients coexisted with hypertension. Patients with severe COVID-19 with hypertension were more likely to be older (median [IQR], 68.5 [60.8-77.0] vs 54.5 [41.0-65.3] years; P < 0.001) and to have previous coronary artery disease (16 of 70 [22.9%] vs 6 of 150 [4.0%]; P < 0.001), and were more likely to be exhibit dyspnea (38 of 70 [54.3%] vs 56 of 150 [37.3%]; P < 0.001) and have a higher systolic blood pressure (median [IQR], 130 [122-144] vs 121 [115-131] mmHg; P = 0.001) at admission (Table 1). There was no significant discrepancies in other signs and symptoms between the two groups.

**Table 1:** Baseline characteristics of patients infected with severe COVID-19.

**Characteristics**	**All patients (n = 220)**	**Hypertension (n = 70)**	**Control (n = 150)**	***P trend***
Age, years	59.5 (48.3-70.0)	68.5 (60.8-77.0)	54.5 (41.0-65.3)	< 0.001
<60	110 (50)	16 (22.9)	94 (62.7)	
≥60, <75	69 (31.4)	31 (44.3)	38 (25.3)	
≥75	41 (18.6)	23 (32.8)	18 (12.0)	
Sex				
Male	106 (48.2)	38 (54.3)	68 (45.3)	0.132
Female	114 (51.8)	32 (45.7)	82 (54.7)	
CAD	22 (10.0)	16 (22.9)	6 (4.0)	< 0.001
Diabetes mellitus	34 (15.5)	14 (20.0)	20 (13.3)	0.203
CVD	8 (4.5)	4 (6.5)	4 (3.5)	0.371
COPD	8 (3.6)	3 (4.3)	5 (3.3)	0.770
Malignancy	4 (1.8)	2 (2.9)	2 (1.3)	0.431
Chronic liver disease	7 (3.2)	1 (1.4)	6 (4.0)	0.312
**Signs and symptoms**				
Fever	173 (78.6)	56 (80.0)	117 (78.0)	0.599
Fatigue	85 (38.6)	30 (42.9)	55 (36.7)	0.380
Cough	146 (66.4)	44 (62.9)	102 (68.0)	0.452
Myalgia	22 (10.0)	5 (7.1)	17 (11.3)	0.335
Dyspnea	94 (42.7)	38 (54.3)	56 (37.3)	0.018
Pharyngalgia	15 8.5)	2 (3.2)	13 (11.4)	0.064
Diarrhea	28 (12.7)	10 (14.3)	18 (12.0)	0.636
Nausea or vomiting	25 (11.4)	7 (10.0)	18 (12.0)	0.688
Headache	11 (5.0)	3 (4.3)	8 (5.3)	0.740
Temperature, IQR	36.7 (36.5-37.1)	36.8 (36.5-37.6)	36.7 (36.5-37.0)	0.476
HR, bpm, IQR	85 (78-95)	84 (77-94)	86 (78-97)	0.568
Respiratory rate, IQR	20 (19-22)	20 (19-25)	20 (19-21)	0.344
SBP (mmHg), IQR	125 (115-135)	130 (122-144)	121 (115-131)	0.001
DBP (mmHg), IQR	78 (70-82)	79 (70-88)	77 (70-81)	0.080

ACE/ARB: Angiotensin-converting enzyme inhibitors/angiotensin receptor blockers, CAD: Coronary artery disease, CDOP: Chronic obstructive pulmonary disease, CVD: Cerebrovascular disease, DBP: Diastolic blood pressure, SBP: Systolic blood pressure, IQR: interquartile range, HR: Heart rate.

In terms of laboratory findings, patients with hypertension compared with patients without hypertension showed higher median creatinine kinase–myocardial band (CK-MB, median [IQR], 2.04 [1.02-3.61] ng/mL vs 0.95 [0.60-2.12] ng/mL), high-sensitivity cardiac troponin I (hs-cTnI, median [IQR], 17 [0-71] pg/ml vs <6 pg/ml), N-terminal pro-B-type natriuretic peptide (median [IQR], 537.8 [172.6-1340.5] pg/mL vs 120.2 [36.7-391.9] pg/mL), white blood cell count (median [IQR], 6260 [4400-8200] cells/μL vs 5170 [3950-6700] cells/μL), and levels of D-dimer (median [IQR], 1.83 [0.56-8.90] mg/L vs 0.61 [0.36-1.98] mg/L), creatinine (median [IQR], 69 [51-84] μmol/L vs 59 [49-72] μmol/L) and lower estimated glomerular filtration rate (eGFR) (median [IQR], 89.3 [58.3-99.1] μmol/L vs 103.9 [91.5-118.0] μmol/L) at admission (Supplementary Table 1).

### Treatment and clinical outcomes

In this cohort, patients with hypertension vs those without hypertension had a similar durations from symptom onset to admission (median [IQR], 11 [8–15] days vs 10 [7–13] days; P = 0.102). Compared with those without hypertension, patients with hypertension required more noninvasive ventilation (23 [32.9%] vs 25 [16.7%]; P = 0.007) and invasive mechanical ventilation (13 [18.6%] vs 13 [8.7%]; P = 0.03). The use of antiviral treatment (63 [90.0%] vs 142 [94.7%]), antibiotic treatment (50 [71.4%] vs 117 [78.0%]), glucocorticoids (32 [43.7%] vs 64 [42.7%]), and intravenous immunoglobulin treatment (32 [51.6%] vs 55 [48.2%]) were similar between patients with hypertension and without hypertension (Table 2). Acute cardiac injury (22 [31.4%] vs 10 [6.7%]; P < 0.001), ARDS (18 [25.7%] vs 13 [8.7%]; P < 0.001) and acute kidney injury (12 [17.1%] vs 7 [4.7%]; P < 0.001) were more common among patients with hypertension than those without hypertension (Table 2).

**Table 2:** Treatments and clinical outcomes in patients with severe COVID-19.

**Treatments and outcomes**	**All patients (n =220)**	**Hypertension (n = 70)**	**Control (n = 150)**	***P trend***
**Treatment**				
Onset of symptom to hospital admission, IQR	10 (7-14)	11 (8-15)	10 (7-13)	0.102
Antiviral treatment	205 (93.2)	63 (90.0)	142 (94.7)	0.201
Antibiotics	167 (75.9)	50 (71.4)	117 (78)	0.288
Glucocorticoid therapy	96 (43.6)	32 (45.7)	64 (42.7)	0.671
Immunoglobulin therapy	87 (49.3)	32 (51.6)	55 (48.2)	0.201
Oxygen inhalation	190 (86.4)	67 (95.7)	123 (82.0)	0.006
NIV	48 (21.8)	23 (32.9)	25 (16.7)	0.007
IMV	26 (11.8)	13 (18.6)	13 (8.7)	0.030
**Outcome**				
Acute cardiac injury	32 (14.5)	22 (31.4)	10 (6.7)	< 0.001
ARDS	31 (14.1)	18 (25.7)	13 (8.7)	< 0.001
Acute kidney injury	19 (8.6)	12 (17.1)	7 (4.7)	< 0.001
In-hospital mortality	39 (17.7)	26 (37.1)	13 (8.7)	< 0.001

ARDS: Acute respiratory distress syndrome; NIV: Noninvasive ventilation; IMV: Invasive mechanical ventilation; IQR: Interquartile range.

### Hypertension and in-hospital mortality

**Figure 1:**
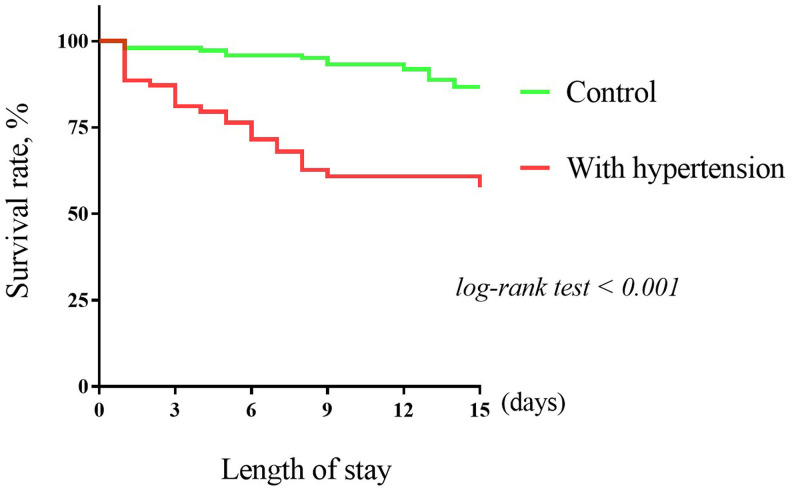
**The Kaplan–Meier survival curves for hypertension and in-hospital mortality.**

**Figure 2:**
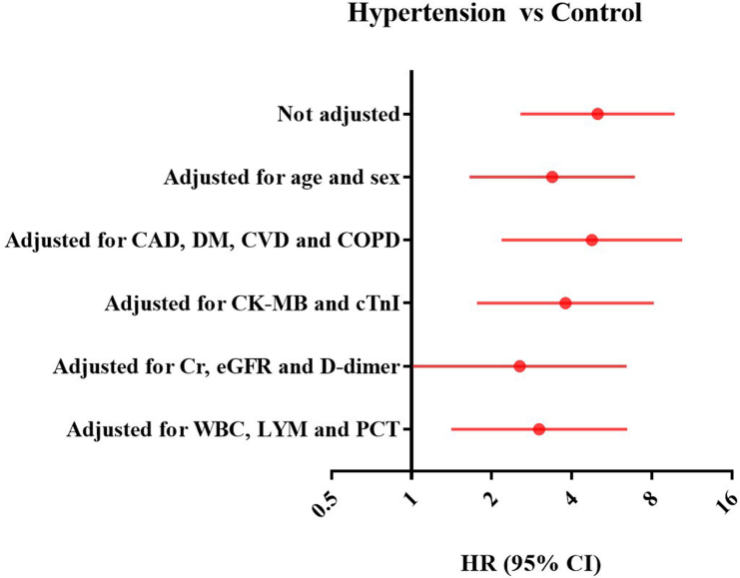
**Forest plots of multivariate Cox proportional-hazards regression analyzing the effect of baseline variables on mortality.** CAD: Coronary artery disease, CI: Confidence interval; CK-MB: Creatinine kinase–myocardial band; COPD: Chronic obstructive pulmonary disease; CVD: Cerebrovascular disease; DM: Diabetes mellitus; eGFR: Estimated glomerular filtration rate; HTN: hypertension; HR: Hazards ratio; hs-cTnI: High-sensitivity cardiac troponin I; LYM: Lymphocytes; PCT: Procalcitonin; Cr: Creatinine, WBC: White blood cell.

There were 39 (17.7%) patients died during hospitalization. The risk of in-hospital mortality was higher among patients with vs without hypertension (26 [37.1%] vs 13 [8.7%]; HR: 5.01; 95%CI: 2.57-9.76, P < 0.001) (Figure 1). To detect potential confounders of in-hospital mortality, we conducted univariate Cox proportional-hazards regression analysis. The results of univariate Cox proportional-hazards regression was shown in Supplementary Table 2. Then, multivariate Cox proportional-hazards regression analyzing was performed to evaluate the effect of baseline variables on mortality (Supplementary Table 3). After full adjusting for potential confounders in Model 1-5, the risk of in-hospital mortality in patients with hypertension was still significantly higher than patients without hypertension (Figure 2).

### ACEI/ARB treatment in severe COVID-19 with hypertension

Of the 70 patients with hypertension, 23 (32.9%) patients received ACEI/ARB (ACEI: 8, ARB: 15) treatment and 47 (67.7%) did not receive ACEI/ARB treatment. Eighteen of 21 patients taken ACEI/ARB before admission and continued during hospitalization, and 2 patients administrated ARB at admission. Most of patients (38 [80.9%]) taken calcium channel blockers in control group. As presented in Supplementary Table 4, there were no significant differences in age, sex, preexisting diseases, signs and symptoms, and blood pressure level between the with ACEI/ARB group and without ACEI/ARB group. However, patients with ACEI/ARB treatment compared with patients without ACEI/ARB treatment showed lower median hs-cTnI (median [IQR], 12 [0-54] pg/mL vs 21 [0-170] pg/mL) and CK-MB (median [IQR], 1.52 [0.81-2.40] ng/ml vs 2.31 [1.23-4.00)] ng/ml) (Supplementary Table 5).

As shown in Table 3, patients treated with ACEI/ARB vs those treated without ACEI/ARB had similar durations from symptom onset to admission (median [IQR], 11 [9-14] days vs 10 [7–15] days; P = 0.893) and treatments (antiviral, antibiotics, glucocorticoid, immunoglobulin and noninvasive or invasive ventilation). Although there was no statistical difference, ACEI/ARB use was associated with a trend for reducing the risk of acute heart injury (4 [17.4%] vs 18 [38.3%]; P = 0.078). No significant differences were observed in the risk of ARDS (6 [26.1] vs 12 [25.5%]; P = 0.960), acute kidney injury (2 [8.7%] vs 10 [21.3%]; P = 0.190) and in-hospital mortality (6 [26.1%] vs 20 [42.6%]; P = 0.181) between the two groups.

**Table 3:** Treatments and clinical outcomes in severe COVID-19 with hypertension.

**Treatments and outcomes**	**ACEI/ARB(n = 23)**	**no ACEI/ARB (n = 47)**	***P trend***
**Treatment**			
Onset of symptom to hospital admission, IQR	11 (9-14)	10 (7-15)	0.893
Antiviral treatment	21 (91.3)	42 (89.4)	0.799
Antibiotics	16 (69.6)	34 (72.3)	0.809
Glucocorticoid therapy	13 (56.5)	19 (40.4)	0.072
Immunoglobulin therapy	13 (65.0)	19 (45.2)	0.146
Oxygen inhalation	22 (95.6)	43 (91.5)	0.985
NIV	8 (34.8)	15 (31.9)	0.810
IMV	5 (21.7)	8 (17.0)	0.634
**Outcome**			
Acute cardiac injury	4 (17.4)	18 (38.3)	0.078
ARDS	6 (26.1)	12 (25.5)	0.960
Acute kidney injury	2 (8.7)	10 (21.3)	0.190
In-hospital mortality	6 (26.1)	20 (42.6)	0.181

ACEI/ARB: Angiotensin-converting enzyme inhibitors/angiotensin receptor blockers; ARDS: Acute respiratory distress syndrome; NIV: Noninvasive ventilation; IMV: Invasive mechanical ventilation; IQR: Interquartile range.

## DISCUSSION

The major findings of our study are the following: (i) hypertension is highly prevalent among patients with severe COVID-19; (ii) patients with hypertension were more likely to suffer from severe inflammatory and acute cardiac injury; (iii) hypertension is an independent risk factor of in-hospital mortality; (iv) the use of ACEI/ARB may protect COVID-19 from acute cardiac injury.

Several epidemiological studies have reported that hypertension is one of the most distinctive comorbidities in patients with severe COVID-19 (approximately 23-56.6%) [4–5, 11]. Consistent with these studies, our data showed that 31.8% patients with severe COVID-19 had a history of hypertension. Patients with severe COVID-19 with hypertension seemed to be older and have more comorbidities than those without hypertension. In this study, we found that patients with hypertension were more likely to suffer from acute cardiac injury.

It might be explained by the reason that the proportion of patients with coronary artery disease in hypertension was higher than patients without hypertension, and more potential cardiac dysfunction might preexist in patients with hypertension, such as left ventricular hypertrophy and heart failure. This may also be one of the reasons why dyspnea was more common in patients with hypertension. Recently, some studies reported that cardiac injury was a frequent condition among hospitalized patients with COVID-19, and it was associated with a higher risk of in-hospital mortality [12].

We have also observed that patients with hypertension suffered from more severe inflammation than cases without hypertension. The expression of ACE2 is relatively insufficient in hypertension. Persistent severe acute respiratory syndrome coronavirus (SARS-CoV-2) infection and replication contribute to reduced ACE2 expression [13–14]. The down-regulation of ACE2 activity in the organs promotes the initial leukocytes infiltration, which contributes to the extent of inflammation after viral infection [15]. In addition, the accelerated ageing of the immune system in hypertension may be another reason why hypertensive patients are potentially related to a more severe inflammation in COVID-19 [16].

Although hypertension has become a major comorbidity in patients with COVID-19, it is unclear whether hypertension is directly related to an increased risk of fatal outcomes. In our study, we found that hypertension was associated with a significantly high risk of in-hospital mortality. Hypertension plays a key role in the development of chronic target organ damage (mainly heart and kidney) and may be exacerbated after SARS-CoV-2 infection, just like other infections. These conditions may lead to higher hospital mortality.

Even if the HR value was weakened after adjustment for multiple potential confounders, including sex, age, cardiac injury, inflammation makers and emerging risk factors of renal dysfunction and D-dimer [17–18], hypertension is still an independent risk factor of in-hospital mortality. In addition, we found that the risk of ARDS in hypertensive patients was significantly higher than that in non- hypertensive patients. The aggravated inflammation in hypertension may contribute to the increased risk of ARDS.

A previous study reported that ACEI/ARB chronic therapy, through their beneficial effects on the cardiovascular system, had a positive effect on survival in very elderly hospitalized patients [19]. Although there are few reports in humans regarding the effects of ACEI/ARB on ACE2 expression [20, 21], experimental evidence has supported that the expression of ACE2 is substantially up-regulation by administering ACEI/ARB [22]. Based on this effect, the use of ACEI/ARB has been proved to be effective in reducing lung injury in animal models of ARDS [23]. However, it has been confirmed that SARSCoV-2 uses the ACE2 receptor as an entry point into the host cell [24]. Therefore, the use of ACEI/ARB in COVID-19 with hypertension were controversial.

Fang et al suggested that hypertension with ACE2-stimulating drugs was associated with an increased risk of developing severe and fatal COVID-19 [9]. But several recent studies have reported that the use of ACEI/ARB has nothing to do with the increased risk of fatal consequences of COVID-19 combined with hypertension [4, 25, 26]. In agreement with these studies, we also found that the use of ACEI/ARB did not have adverse effect on signs, symptoms and clinical outcomes in severe COVID-19. Interestingly, we found that the ACEI/ARB treatment was associated with lower level of hs-cTnI, CK-MB and a lower risk of acute cardiac injury, which indicated that ACEI/ARB use might help to alleviate cardiac injury of severe COVID-19. The benefits of ACEI/ARB on cardiovascular diseases may help reduce the risk of acute cardiac injury [19, 27].

This study has several limitations that must be acknowledged. First, as the data on echocardiography was waived in light of the urgent medical condition, the underlying cardiac function of severe COVID-19 cases was unclear. We could not entirely rule out the possibility of residual confounders by these unmeasured parameters. Second, data were extracted from a pre-existing electronic medical records, which subjects it to potential selection bias. But it could be minimized by conducting multivariate regression analyses. Third, for the effect of ACEI/ARB on severe COVID-19 with hypertension, the sample size of this study was small and our findings still need to be further confirmed.

In summary, patients with COVID-19 with hypertension was more likely to suffer from severe cardiac injury, inflammatory response and poor prognosis. Considering the high risk of death, incremental caution and in-hospital discussion of anticipated risk in hypertension are completely necessary. In addition, administration of ACEI/ARB may contribute to low risk of acute cardiac injury in severe COVID-19. Our findings supported that ACEI/ARB can continue to be used in patients with severe COVID-19.

## MATERIALS AND METHODS

### Study design and participants

This was a retrospective, observational study registry with https://clinicaltrials.gov/ identifier NCT04292964. Consecutive patients with severe COVID-19 admitted to hospitals in Hubei and Chongqing from January 11st, 2020 to February 20^th^, 2020, were collected by national medical team. Case definitions of confirmed human infection with COVID-19 conformed to the interim guidance from the World Health Organization. All patients were diagnosed as severe cases according to the 7^th^ edition guideline issued by China’s National Health Commission. This study was approved by the institutional ethics board of the First Affiliated Hospital of Chongqing Medical University (approval NO. 20200701). The study was also registered on Chinese medical research registration information system.

### Data collections

Information on symptoms, signs, underlying comorbidities, laboratory findings, blood pressure, medical history (i.e. ACEI, ARB and calcium channel blocker), treatments and clinical outcomes were obtained from electronic medical records. All the data were collected by two investigators independently and double checked by other investigators. If the information on medical history were not available from electronic medical records, we directly communicated with patients or their families to obtain further data.

### Definition and study outcome

Severe COVID-19 was defined as meeting at least one of the following criteria: 1) respiratory rate ≥30/min; 2) at rest, oxygen saturation (SpO_2_) is ≤ 93%; 3) partial pressure of oxygen (PaO_2_)/Fraction of inspiration O_2_ (FiO_2_) ≤ 300mmHg. ACEI/ARB treatment was defined as a patient taking ACEI/ARB during hospitalization irrespective of dose and whether taken before admission or not. The primary endpoint was in-hospital mortality, defined as any death occurring within 15 days after hospitalization. The secondary endpoints included acute cardiac injury, ARDS and acute kidney injury. Acute cardiac injury was defined as blood levels of cardiac biomarkers (hs-cTnI) above the 99^th^-percentile upper reference limit.

### Statistical analysis

Continuous measurements described as mean (standard deviation: [SD]) if they are normally distributed or median (interquartile range: [IQR)] if they are not. Categorical variables were described as frequency rates and percentages. Test for differences in categorical variables among the two groups used X^2^ test. T test or Mann-Whitney test was used to compare continuous variables according to the normal distribution or not. The Kaplan–Meier product-limit estimation method was used to estimate survival rates, and the statistical differences were compared using log-rank tests. The univariate and multivariate Cox proportional hazard model was used to estimate the hazard ratios (HRs) and 95% confidence intervals (CIs). All P values were two-tailed, and significance was set at P < 0.05. Statistical analyses were performed using SPSS software (version 22.0).

## Supplementary Material

Supplementary Tables
