# Supplementary material for: Clinical characteristics and fatal outcomes of hypertension in patients with severe COVID-19
Source: Aging (Albany NY). 2020 Nov 16;12(23):23436–49. doi: 10.18632/aging.104019 (PMC7762496; doi:10.18632/aging.104019)
Supplement: Supplementary Tables [file aging-12-104019-s001.pdf]

SUPPLEMENTARY TABLES

Supplementary Table 1. Laboratory findings of patients infected with severe COVID-19 at admission.

Variables, IQR or %	Normal Range	All patients (n = 220)	Hypertension (n = 70)	Control (n = 150)	*P trend*
hs-cTnI, pg/mL	0-40	4 (0-19)	17 (0-71)	0 (0-9)	< 0.001
CK–MB, ng/mL	0-5	1.14 (0.67-2.69)	2.04 (1.02-3.61)	0.95 (0.60-2.12)	< 0.001
NT-proBNP, pg/mL	> 90	220.4 (52.3-867.1)	537.8 (172.6-1340.5)	120.2 (36.7-391.9)	< 0.001
Total cholesterol, mmol/L	0-5.2	3.70 (3.29-4.33)	3.85 (3.34-4.26)	3.66 (3.28-4.48)	0.987
LDL cholesterol, mmol/L	0-3.4	2.4 (1.9-2.9)	2.40 (1.87-2.45)	2.24 (1.85-2.81)	0.623
HDL cholesterol, mmol/L	>1.00	0.90 (0.76-1.06)	0.88 (0.74-1.00)	0.92 (0.78-1.06)	0.282
Triglycerides, mmol/L	0-1.7	1.27 (0.99-1.80)	1.19 (0.99-1.60)	1.33 (0.96-1.99)	0.370
WBC count, $\times10^9$/L	3.5-9.5	5.45 (4.10-7.05)	6.26 (4.44-8.20)	5.17 (3.95-6.70)	0.003
Neutrophil count, $\times10^9$/L	1.8-6.3	3.74 (2.58-5.49)	4.48 (2.84-6.77)	3.33 (2.41-4.89)	0.002
hs-CRP, mg/L	0-3	39.80 (5.83-87.50)	56.50 (15.50-128.60)	25.90 (3.77-64.30)	< 0.001
Procalcitonin, ng/mL	0-1.0	0.06(0.03-0.14)	0.08 (0.03-0.19)	0.06 (0.03-0.12)	0.061
Lymphocyte count, $\times10^9$/L	1.1-3.2	1.02 (0.71-1.43)	0.92 (0.55-1.43)	1.05 (0.75-1.43)	0.193
Monocyte count, %	3--10	8.20 (5.20-10.45)	7.00 (3.40-9.50)	8.45 (5.93-10.70)	0.044
Platelet count, $\times10^9$/L	125-350	198 (147-261)	199 (139-278)	197 (147-260)	0.726
Prothrombin time, s	9--13	11.90 (11.30-12.60)	12.30(11.60-12.90)	11.50 (11.13-12.50)	< 0.001
APTT, s	25-31.3	27.90 (25.80-31.25)	27.70 (25.80-30.90)	27.90 (25.75-31.28)	0.996
D-dimer, mg/L	0-0.55	0.80 (0.40-3.80)	1.83 (0.56-8.90)	0.61 (0.36-1.98)	< 0.001
ALT, U/L	7-40	26 (16-42)	25 (17-50)	27 (16-40)	0.398
AST, U/L	13-35	30 (20-43)	32 (21-50)	28 (20-41)	0.036
Total bilirubin, mmol/L	0-23	10.30 (7.50-15.45)	10.35 (7.25-16.20)	10.10 (7.50-15.20)	0.804
Blood urea nitrogen, mmol/L	3.1-8.8	4.70 (3.53-6.47)	5.73 (4.15-9.30)	4.36 (3.39-5.53)	< 0.001
Creatinine, µmol/L	41-81	61 (49-75)	69 (51-84)	59 (49-72)	0.023
eGFR	> 90	98.8 (82.0-109.5)	89.3 (57.3-99.1)	103.9 (91.5-118.0)	< 0.001
Bilateral distribution of patchy shadows or ground glass opacity	—	215 (97.7)	69 (98.6)	148 (98.7)	0.955

ALT: Alanine aminotransferase; AST: Aspartate aminotransferase, APTT: Activated partial thromboplastin time, CK-MB: creatinine kinase–myocardial band, eGFR: Estimated glomerular filtration rate, HDL: High density lipoprotein, hs-CRP: High-sensitivity C-reactive protein, hs-cTnI: high-sensitivity cardiac troponin I, IQR: interquartile range, LDL: Low density lipoprotein, NT-proBNP: N-terminal pro-B-type natriuretic peptide, WBC: White blood cell.

Supplementary Table 2. Univariate Cox proportional-hazards regression analyzing the effect of baseline variables on in-hospital mortality.

Characteristics		HR (95%CI)	P-value
Sex			
Male		2.11 (1.09-4.05)	0.026
Female			
Age, ≥65 years		4.20 (2.09-8.44)	<0.001
Hypertension	Yes	5.01 (2.57-9.76)	<0.001
	No		
History of DM	Yes	1.41 (0.65-306)	0.391
	No		
History of CAD	Yes	2.55 (1.17-5.55)	0.019
	No		
History of COPD	Yes	2.75 (0.98-7.73)	0.056
	No		
History of CVD	Yes	3.75 (1.31-10.71)	0.013
	No		
CK-MB, >5ng/ml		1.79 (0.83-3.86)	0.140
hs-cTnI, >1ng/ml		10.06 (4.26-23.76)	<0.001
D-dimer, >1 mg/L		7.2 (2.74-18.51)	<0.001
Creatinine, >81umol/L		4.38 (2.19-8.76)	<0.001
eGFR, <90mL/(min·1.73 m^2)		4.66 (2.21-9.80)	<0.001
WBC, >9.3×10^9/L		6.12 (3.11-12.06)	<0.001
LYM, <1.1×10^9/L		5.23 (2.03-13.49)	0.001
PCT, >1ng/ml		6.50 (3.10-13.63)	<0.001

CAD: Coronary artery disease; CI: Confidence interval; CK-MB: Creatine kinase-MB; COPD: Chronic obstructive pulmonary disease; CVD: Cerebrovascular disease; DM: Diabetes mellitus; eGFR: Estimated glomerular filtration rate; HR: Hazards ratio; hs-cTnI: High-sensitivity cardiac troponin I; LYM: Lymphocytes; PCT: Procalcitonin; WBC: White blood cell.

Supplementary Table 3. Multivariate Cox proportional-hazards regression analyzing the effect of baseline variables on in-hospital mortality.

Mode	HR (95%CI)	P-value
Not Adjusted	5.01 (2.57-9.76)	<0.001
**Mode 1**		
Hypertension	3.38 (1.65-6.92)	0.001
Age ≥65years	2.65 (1.25-5.60)	0.011
Male vs female	2.00 (1.04-3.86)	0.038
**Mode 2**		
Hypertension	4.77 (2.18-10.43)	<0.001
History of CVD	2.30 (0.75-7.03)	0.144
History of CAD	0.97 (0.35-2.68)	0.958
History of DM	1.25 (0.51-3.06)	0.634
History of COPD	3.98 (0.97-16.32)	0.055
**Mode 3**		
Hypertension	3.79 (1.76-8.16)	0.001
CK-MB, >5ng/ml	1.64 (0.75-3.58)	0.235
hs-cTnI, >1ng/ml	5.69 (2.33-13.91)	<0.001
**Mode 4**		
Hypertension	2.55 (1.01-6.45)	0.048
D-dimer, >1 mg/L	3.81 (1.43-10.18)	0.008
Creatinine, >100 umol/L	6.21 (1.59-24.18)	0.009
eGFR, <60mL/(min·1.73 m^2)	0.69 (0.17-2.82)	0.602
**Mode 5**		
Hypertension	3.02 (1.41-6.47)	0.005
WBC, >9.3×10^9/L	3.03 (1.40-6.58)	0.005
LYM, <1.1×10^9/L	3.24 (1.20-8.77)	0.020
PCT, >1ng/ml	2.63 (1.17-5.91)	0.019

CAD: Coronary artery disease; CI: Confidence interval; CK-MB: Creatine kinase-MB; COPD: Chronic obstructive pulmonary disease; CVD: Cerebrovascular disease; DM: Diabetes mellitus; eGFR: Estimated glomerular filtration rate; HR: Hazards ratio; hs-cTnI: High-sensitivity cardiac troponin I; LYM: Lymphocytes; PCT: Procalcitonin; WBC: White blood cell.

Supplementary Table 4. Baseline characteristics of hypertension infected with severe COVID-19.

Variable	ACEI/ARB (n = 23)	No ACEI/ARB (n = 47)	*P trend*
Age, y IQR	69 (59-75)	68 (62-78)	0.417
<60	6 (26.1)	10 (21.3)	
≥60, <75	10 (43.5)	21 (44.7)	
≥75	7 (30.4)	16 (34.0)	
Sex			
Male	10 (43.5)	28 (59.6)	0.204
Female	13 (56.5)	19 (40.4)	
CAD	5 (21.7)	11 (23.4)	0.876
Diabetes mellitus	4 (17.4)	12 (25.5)	0.446
CVD	0 (0)	4 (8.5)	0.150
COPD	1 (4.3)	2 (4.3)	0.986
Malignancy	0 (0)	2 (4.3)	0.316
Chronic liver disease	0 (0)	0 (0)	>0.99
**Signs and symptoms**			
Fever	18 (90.0)	33 (78.6)	0.715
Temperature, IQR	36.7 (36.4-37.5)	36.8 (36.5-37.6)	0.671
Fatigue	9 (45.0)	15 (38.1)	0.483
Cough	13 (65.0)	26 (61.9)	0.814
Myalgia	1 (5.0)	2 (4.8)	0.967
Dyspnea	9 (45.0)	22 (52.4)	0.587
Pharyngalgia	1 (5.0)	1 (2.4)	0.585
Diarrhea	3 (15.0)	4 (9.5)	0.524
Nausea or vomiting	2 (10.0)	2 (4.8)	0.433
Headache	1 (5.0)	2 (4.8)	0.967
HR, bpm, IQR	84 (76-92)	85 (78-95)	0.508
Respiratory rate, IQR	20 (19-21)	20 (19-28)	0.116
SBP (mmHg), IQR	132 (127-140)	129 (116-150)	0.291
DBP (mmHg)	78 (70-85)	80 (69-90)	0.501

ACEI/ARB: Angiotensin-converting enzyme inhibitors/angiotensin receptor blockers, CAD: Coronary artery disease, CDOP: Chronic obstructive pulmonary disease, CVD: Cerebrovascular disease, DBP: Diastolic blood pressure, SBP: Systolic blood pressure, IQR: interquartile range, HR: Heart rate.

Supplementary Table 5. Laboratory findings of hypertension infected with severe COVID-19 at admission.

Variables, IQR or %	ACEI/ARB (n = 23)	No ACE/ARB (n = 47)	*P trend*
hs-cTnI, pg/mL	12 (0-54)	21 (0-170)	0.019
CK–MB, ng/mL	1.52 (0.81-2.40)	2.31 (1.23-4.00)	0.021
NT-proBNP, pg/mL	477 (222-1634)	609 (127-1290)	0.678
Total cholesterol, mmol/L	2.39 (1.88-2.87)	3.69 (3.35-4.36)	0.668
LDL cholesterol, mmol/L	2.40 (1.87-2.45)	2.41 (1.97-2.74)	0.989
HDL cholesterol,mmol/L)	0.91 (0.79-1.08)	0.86 (0.71-0.99)	0.193
Triglycerides (mmol/L)	1.55 (0.98-1.74)	1.19 (1.01-1.57)	0.403
WBC count, $\times 10^9$/L	6.50 (4.41-8.50)	6.15 (4.45-8.12)	0.692
Neutrophil count, $\times 10^9$/L	4.50 (3.12-7.85)	4.40 (2.72-6.62)	0.506
hs-CRP, mg/L	57.8 (7.5-135.5)	53.4 (18.9-102.9)	0.921
Procalcitonin, ng/mL	0.075 (0.038-0.220)	0.091 (0.032-0.184)	0.921
Lymphocyte count, $\times 10^9$/L	0.90 (0.65-1.18)	1.08 (0.54-1.50)	0.468
Monocyte count, (%)	6.90 (3.55-8.73)	7.60 (3.40-10.10)	0.659
Platelet count, $\times 10^9$/L	210 (157-290)	192 (133-274)	0.492
Prothrombin time, s	12.40 (11.83-12.80)	12.10 (11.60-13.10)	0.899
APTT, s	27.6 (24.8-30.7)	27.7 (26.2-32.8)	0.256
D-dimer, mg/L	1.42 (0.51-4.93)	1.96 (0.66-15.90)	0.350
ALT, U/L	26 (17-59)	25 (16-51)	0.747
AST, U/L	32 (20-45)	32 (22-51)	0.568
Total bilirubin, mmol/L	14.00 (10.30-18.75)	8.90 (6.10-15.20)	0.004
Blood urea nitrogen, mmol/L	5.90 (4.45-8.69)	5.50 (3.80-10.90)	0.952
Creatinine, µmol/L	65.0 (56.5-75.0)	70.0 (48.0-112.0)	0.304
eGFR	90.5 (65.3-7-95.4)	84.2 (52.2-100.3)	0.868
Bilateral distribution of patchy shadows or ground glass opacity	23 (100)	46 (97.9)	0.481

ACEI/ARB: Angiotensin-converting enzyme inhibitors/angiotensin receptor blockers; ALT: Alanine aminotransferase; AST: Aspartate aminotransferase, APTT: Activated partial thromboplastin time, CK-MB: creatinine kinase–myocardial band, eGFR: Estimated glomerular filtration rate, HDL: High density lipoprotein, hs-CRP: High-sensitivity C-reactive protein, hs-cTnI: high-sensitivity cardiac troponin I, IQR: interquartile range, LDL: Low density lipoprotein, NT-proBNP: N-terminal pro-B-type natriuretic peptide, WBC: White blood cell.
